# DNA barcodes provide insights into the diversity and biogeography of the non‐biting midge *Polypedilum* (Diptera, Chironomidae) in South America

**DOI:** 10.1002/ece3.10602

**Published:** 2023-10-11

**Authors:** Fabio Laurindo da Silva, Luiz Carlos Pinho, Elisabeth Stur, Silvio Shigueo Nihei, Torbjørn Ekrem

**Affiliations:** ^1^ Department of Natural History NTNU University Museum, Norwegian University of Science and Technology Trondheim Norway; ^2^ Laboratory of Systematic of Diptera, Department of Ecology and Zoology Federal University of Santa Catarina Florianópolis Brazil; ^3^ Laboratory of Systematic and Biogeography of Insecta, Department of Zoology, Institute of Biosciences University of São Paulo São Paulo Brazil; ^4^ Present address: Laboratory of Aquatic Insect Biodiversity and Ecology, Department of Zoology, Institute of Biosciences University of São Paulo São Paulo Brazil

**Keywords:** biogeography, Chironomidae, distance‐based methods, DNA barcode, tree‐based methods

## Abstract

South America, particularly within its tropical belt, is renowned for its unparalleled high levels of species richness, surpassing other major biomes. Certain neotropical areas harbor fragmented knowledge of insect diversity and face imminent threats from biodiversity loss and climate change. Hence, there is an urgent need for rapid estimation methods to complement slower traditional taxonomic approaches. A variety of algorithms for delimiting species through single‐locus DNA barcodes have been developed and applied for rapid species diversity estimates across diverse taxa. However, tree‐based and distance‐based methods may yield different group assignments, leading to potential overestimation or underestimation of putative species. Here, we investigate the performance of different DNA‐based species delimitation approaches to rapidly estimate the diversity of *Polypedilum* (Chironomidae, Diptera) in South America. Additionally, we test the hypothesis that significant differences exist in the community structure of *Polypedilum* fauna between South America and its neighboring regions, particularly the Nearctic. Our analysis encompasses a dataset of 1492 specimens from 598 locations worldwide, with a specific focus on South America. Within this region, we analyzed a subset of 247 specimens reported from 37 locations. Using various methods including the Barcode Index Number (BIN), Bayesian Poisson tree processes (bPTP), multi‐rate Poisson tree processes (mPTP), single‐rate Poisson tree processes (sPTP), and generalized mixed Yule coalescent (sGMYC), we identify molecular operational taxonomic units (MOTUs) ranging from 267 to 520. Our results indicate that the sGMYC method is the most suitable for estimating putative species in our dataset, resulting in the identification of 75 species in the Neotropical region, particularly in South America. Notably, this region exhibited higher species richness in comparison to the Palearctic and Oriental realms. Additionally, our findings suggest potential differences in species composition of *Polypedilum* fauna between the Neotropical and the adjacent Nearctic realms, highlighting high levels of endemism and species richness in the first. These results support our hypothesis that there are substantial differences exist in species composition between the *Polypedilum* fauna in South America and the neighboring regions.

## Graphical abstract

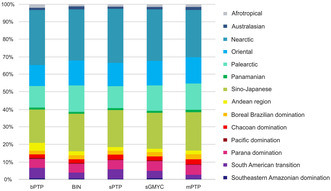

## 1 INTRODUCTION

In the past decades, natural environments have been disturbed and destroyed worldwide at alarming rates, which results in a large loss of species (Barnosky et al., 2011; Dirzo et al., 2014; Stork, 2018). In highly diverse biomes, such as those found in the Neotropical region, the risk of species extinctions occurring prior to their identification is significant (Bradshaw et al., 2009; Laurance, 1999). This indicates that biodiversity evaluation needs to be accelerated by combining the strengths of molecular biology, sequencing technology, and bioinformatics to recognize previously known and described species (Gostel & Kress, 2022) and to allow new findings. In this context, DNA‐based approaches have become increasingly useful and promising tools for estimating diversity and guaranteeing rapid and accurate identification of species. Since the proposal of the DNA barcoding technique, using a short standard genetic marker for species‐level identification and cryptic species detection (Hebert et al., 2003, 2004), the procedure has been becoming progressively popular among conservationists and taxonomists (Chimeno et al., 2022; Farooq et al., 2020; Pearl et al., 2022) and paved the way for biological monitoring using metabarcoding (e.g., Steinke et al., 2022).

Insects with both aquatic and terrestrial phases in the life cycle play a crucial role for the equilibrium of aquatic ecosystems because of their complex life cycle, which distinguishes them from exclusively aquatic or terrestrial life forms, and generates a differentiated potential for understanding biogeographical and ecological research (Suter & Cormier, 2015). It is therefore paramount to invest in knowledge of the diversity of these organisms, as they are extremely rich both in functionality and species numbers. Non‐biting midges (Diptera: Chironomidae) are true flies and frequently dominate aquatic insect communities in both abundance and species richness. It is a cosmopolitan group, occurring in an enormous variety of aquatic, semiaquatic, and terrestrial ecosystems, in all biogeographical regions of the world, including Antarctica (Armitage et al., 1995). Presumably, the great species and habitat diversity in this family is a product of its early radiation, relatively low vagility, and evolutionary plasticity (Cranston et al., 2012; Ferrington Jr., 2008), which makes the family not only a valuable source of bioindicator species for assessing the health of lentic and lotic aquatic ecosystems but also one of the most intriguing groups for phylogenetic and biogeographical analyses (Silva & Ekrem, 2016).

The genus *Polypedilum* is one of the largest chironomid genera, comprising more than 500 described species (Song et al., 2018). This taxonomic richness and unique characteristics make it an interesting subject for biodiversity research. The larvae of *Polypedilum* occur in nearly all types of still and flowing waters. Morphological identification of *Polypedilum* species can be challenging, leading to difficulties in taxonomic studies. Additionally, the limited association between immature and adult stages further complicates the understanding of their life cycles. Despite these challenges, the ecological and biogeographical aspects of *Polypedilum* are of great interest due to their wide distribution and substantial species richness. While taxonomic or phylogenetic studies on *Polypedilum* are abundant (e.g., Bidawid & Fittkau, 1995; Bidawid‐Kafka, 1996; Oyewo & Sæther, 2008; Pinho & Silva, 2020; Sæther & Oyewo, 2008; Sæther & Sundal, 1999; Sæther et al., 2010; Shimabukuro et al., 2019; Vårdal et al., 2002), detailed investigations on the species richness and species turnover within the hyperdiverse Chironomidae, including *Polypedilum*, have been limited (Lin et al., 2015; Song et al., 2018).

Dealing with hyperdiverse taxa such as *Polypedilum* offers distinct advantages, as they often exhibit multiple recurring patterns that can provide evidence of underlying processes (Coscarón et al., 2009). These patterns can encompass various aspects, including iterative characteristics, recurrent biogeographical distributions, and replicated ecological traits. For instance, one notable example is the reiterated occurrence of the transantarctic distribution observed in different groups or species within the Chironomidae family, as described by Brundin (1966). These recurrent patterns offer valuable insights into the evolutionary dynamics, ecological interactions, and biogeographical factors that contribute to the diversity and distribution of hyperdiverse taxa like *Polypedilum*. By analyzing and understanding these iterative patterns, the mechanisms driving the remarkable diversity and distribution of such taxa can unraveled, contributing to our broader understanding of biodiversity and ecosystem processes.

The biota of South America always has attracted the attention of naturalists because of the interesting distributional patterns exhibited by its fauna and flora (Brundin, 1966; Darwin, 1859; Hooker, 1844; Wallace, 1876). For more than a century, biogeographers have proposed theories to explain the origin and relationships of the biodiversity found in South America and other southern temperate regions such as Australia, New Zealand, and South Africa (Silva & Farrell, 2017). Moreover, the region is a preferred target for investigating the function of these components in the dynamic of diversification, both by harboring the majority of the Earth's species and extending across temperate and tropical belts. The high number of species in South America, on a regional as well as on a continental scale, makes the region an important reference mark for estimation of biodiversity loss. However, the Neotropical non‐biting midge fauna presents an incomplete understanding of actual species diversity, often making formal identifications unachievable (Spies & Reiss, 1996).

This fragmented knowledge poses challenges in unraveling the mechanisms driving the hyperdiversity observed in the Neotropics. To overcome these challenges, automated species delimitation approaches have emerged as valuable tools. These methods utilize molecular data to explore species boundaries in organisms with uncertain taxonomic knowledge or when signals in phylogenetic inferences are obscured by lineage sorting or introgression (O'Meara, 2010 and references therein). In this sense, several methods for species delimitation have been developed and applied, for instance, the Automatic Barcode Gap Discovery – ABGD (Puillandre et al., 2012), the Barcode Index Number – BIN (Ratnasingham & Hebert, 2013), the Generalized Mixed Yule Coalescent – GMYC (Pons et al., 2006), the Poisson Tree Processes – PTP (Zhang et al., 2013). Despite these approaches being suitable to delimit species, they can occasionally lead to uncertainty in genetic diversity estimates due to either oversplitting or overlumping of the taxa. Therefore, the integration of different algorithms is needed for accurate species delimitation. In this study, we first compare the performance of different methods of species estimation and evaluate how much these different approaches affect estimates of putative species richness in South America. We then test the hypothesis that there will be substantial differences in species composition between the *Polypedilum* fauna in South America, known for its higher diversity, and neighboring regions, particularly the Nearctic.

## 2 MATERIALS AND METHODS

### 2.1 Taxon sampling and data collection

Specimens were collected between 2014 and 2017 from 34 localities, in a diverse array of habitats including small streams and ponds to lakes, rivers, and bays in Argentina, Brazil, Chile, and Dominican Republic. These collections were made by our research team as part of our study on the biogeographical patterns of chironomids in the Neotropical region. The main emphasis was on adult sampling, collected with a sweep net near aquatic systems. A 20 cm diameter D‐frame kick net (mesh size 250 μm) was also used to collect immature stages at some localities. All sampled adults were preserved in 75%–85% ethanol and larvae in 96%–100% ethanol and stored at 4°C in the dark prior to the extraction. Specimens were identified using the classification proposed by Bidawid and Fittkau (1995), Bidawid‐Kafka (1996), Pinho and Silva (2020), Shimabukuro et al. (2019), Townes Jr. (1945), and eventual examination of type material. Voucher specimens are deposited in the Museum of Comparative Zoology (MCZ) at Harvard University and in the National Institute of Amazonian Research (INPA).

In addition to data generated for this publication, we also searched for public COI barcodes in the Barcode of Life Data Systems (BOLD, www.boldsystems.org) belonging to the genus *Polypedilum* that were longer than 300 base pairs and without stop codons. Searches were performed on 25 January 2022 in BOLD. Therefore, out of the 9540 COI barcodes included in our dataset, 149 barcodes of 54 identified species were obtained from specimens collected specifically for this study. These specimens were not previously used in any molecular analysis. A reduced data set, containing 1492 sequences, was generated by the manual deletion of the highly similar sequences based on an UPGMA tree. Identical sequences, which refers to genetic sequences that share extreme similarity in the barcode region, occurring at different sampling localities were retained in our dataset. The detailed specimen records and sequence information, including trace files, are available in BOLD Systems through the dataset ‘DS‐RRPOL – Reduced Records of *Polypedilum* (Diptera: Chironomidae)’ with DOI: https://doi.org/10.5883/DS‐RRPOL.

### 2.2 DNA extraction, PCR amplification, sequencing and alignment

The targeted taxa were sorted and dissected under a stereo microscope. Thorax and one pair of legs were used for genomic DNA extraction. All extraction procedures followed the Qiagen DNeasy Blood and Tissue kit protocol provided by the manufacturer. DNA was extracted in a buffered solution with the enzyme proteinase‐K at 56°C overnight, and otherwise followed the manufacturer's protocol, except using a final elution volume of 100 μL. After digestion, the exoskeleton was removed carefully using a fine‐tipped forceps and washed with 96% ethanol before mounting in Euparal on the same microscope slide as its corresponding head, antennae, wings, legs, and abdomen following the procedure outlined by Sæther (1969).

A 658 bp fragment of the COI region was PCR‐amplified in 25 μL reactions and containing 2 μL DNA template (concentration not measured), 2.5 μL 5X buffer, 2 μL MgCl_2_ in 25 μM concentration, 0.2 μL of dNTPs in 10 mM concentration, 1 μL of each of the universal standard barcode primers (Folmer et al., 1994) LCO1490 (50‐GGTCAACAAATCATAAAGATATTGG‐30) and HCO2198 (50‐TAAACTTCAGGGTGACCAAAAAATCA‐30), in 10 μM concentration, 0.2 μL of HotStarTaq (Qiagen) and 16.1 μL of ddH2O. PCR amplification was performed in a thermocycler with an initial denaturation step of 95°C for 15 min, then followed by five cycles of 94°C for 30 s, 45°C for 30 s, 72°C for 1 min, followed by 35 cycles of 94°C for 30 s, 51°C for 30 s, 72°C for 1 min, and one cycle at 72°C for 5 min, then held at 4°C.

PCR products were checked visually by electrophoresis on a 1.5% agarose gel and purified using shrimp alkaline phosphatase and exonuclease I (USB Corp.). For bidirectional sequencing, we used the ABI PRISM BigDye Terminator version 3.1 Cycle Sequencing Kit (Life Technologies), and cycle sequencing reactions were performed on ABI PRISM 3130xl or 3730xl automated sequencers (Life Technologies) at Harvard University, or shipped to Eurofins Genomics. Raw sequences were assembled and edited using Geneious 2021.2.2 (Kearse et al., 2012), checked for stop codons, and aligned as translated amino acids using the MUSCLE algorithm (Edgar, 2004) on amino acids as implemented in MEGA11 (Tamura et al., 2021). The nucleotide compositions were calculated in MEGA11, while the pairwise genetic distances for each individual sequence were determined in BOLD, both using the K2P model (Kimura, 1980).

### 2.3 Phylogenetic analysis

Two phylogenetic trees were generated: a non‐ultrametric phylogram using Maximum Likelihood (ML) (Felsenstein, 1981) and an ultrametric chronogram using Bayesian inference (BI) (Drummond et al., 2002). The ML tree was generated using Iq‐Tree (Trifinopoulos et al., 2016). Node support was assessed with 1000 ultrafast bootstrap replicates (Hoang et al., 2018), using the GTR + G + I model, with the data partitioned according to codon position, as recommended by PartitionFinder version 2.1. (Lanfear et al., 2017). The BI tree was generated using BEAST version 2.6.7 (Bouckaert et al., 2019), using default settings for all parameters. XML files were made with the BEAUti version 2.6.7 interface with the following settings: GTR + G + I substitution model, empirical base frequencies, 4 gamma categories, and all codon positions partitioned with unlinked base frequencies and substitution rates.

Due to the lack of consensus on the most appropriate clock and tree priors for reconstructing gene trees in species delimitation (Monaghan et al., 2009; Ratnasingham & Hebert, 2013; Talavera et al., 2013; Tang et al., 2014), we conducted a formal test to compare two different clock models (strict and relaxed lognormal) and two different tree priors (coalescent constant population and Yule) (Rodrigues et al., 2020). The aim was to determine the most suitable models for our dataset using the nested sampling (NS) algorithm (Maturana et al., 2019), implemented as a package for BEAST. This was achieved by performing Bayes factor calculations (Kass & Raftery, 1995) using the estimated marginal likelihoods also obtained from BEAST. The results of these exploratory analyses (Table 1) indicated the relaxed clock and Yule priors were the most suitable for our data set. Although the Yule model is a simplistic representation of speciation, assuming a strictly bifurcating pattern of species diversification, it is important to note that a more complex model does not necessarily always provide a better fit to the data just because it seems more biologically realistic (Condamine et al., 2015). Therefore, our decision to employ the Yule prior, along with the relaxed clock, was based on empirical evidence from our dataset (Table 1).

**TABLE 1:** Pairwise comparison of Bayes Factors for molecular clocks and tree prior models in species delimitation analysis.

Model comparison	Bayes factor	Reverse comparison	Bayes factor
Model 1 vs. Model 2	8.1112	Model 2 vs. Model 1	0.1233
Model 1 vs. Model 3	0.0116	Model 3 vs. Model 1	86.1339
Model 1 vs. Model 4	0.0309	Model 4 vs. Model 1	32.3437
Model 2 vs. Model 3	0.0014	Model 3 vs. Model 2	716.8597
Model 2 vs. Model 4	0.0038	Model 4 vs. Model 2	262.6477
Model 3 vs. Model 4	3.6617	Model 4 vs. Model 3	0.2732

*Note*: The Bayes Factors in the ‘Reverse Comparison’ column were calculated by taking the reciprocal of the corresponding Bayes Factor in the ‘Bayes Factor’ column. The Bayes Factors provide a measure of the relative support for different models in species delimitation analysis. A higher Bayes Factor (larger than 1) indicates stronger evidence in favor of a particular model compared to the other. The models used in the analysis are defined as follows: Model 1 (Strict clock + Yule prior), Model 2 (Strict clock + CCP prior), Model 3 (Relaxed clock + Yule prior), and Model 4 (Relaxed clock + CCP prior).

To account for mixing within chains and convergence among chains with reversible jump MCMC (Elworth et al., 2018), a total of 10 chains were run from different seeds for 100 million generations each. Log files from each run were combined in LogCombiner version 2.6.7 (Drummond et al., 2012) after removal of the first 10% of samples from each run as burn‐in. Convergence of each run and the combined data were checked for proper mixing using effective sample size (ESS) > 200 in Tracer version 1.7.1 (Rambaut et al., 2018). Tree files from each run were resampled to retain only 10% of the total trees and combined using LogCombiner after removal of the first 10% of retained trees from each run as burn‐in. A maximum clade credibility (MCC) tree was then produced using TreeAnnotator version 2.6.6 (Drummond et al., 2012) and FigTree version 1.4.4 (Rambaut, 2010) was used to visualize and edit the trees.

All phylogenetic analyses were conducted on the CIPRES Science Gateway High Performance Computing platform (Miller et al., 2011).

### 2.4 Putative species estimation

The two single‐locus DNA barcoding methods consisted of two fundamentally different approaches. First, we implemented three distance‐based approaches: (1) the Automatic Barcode Gap Discovery – ABGD (Puillandre et al., 2012) performed using the online version of the software (https://bioinfo.mnhn.fr/abi/public/abgd/abgdweb.html); (2) the Assemble Species by Automatic Partitioning – ASAP (Puillandre et al., 2021), an updated implementation of the ABGD hierarchical clustering algorithm, performed with the ASAP web version (https://bioinfo.mnhn.fr/abi/public/asap/). Both methods used the MUSCLE‐aligned matrix as the input file and adopted the Kimura model, following the default settings for all parameters. (3) The Barcode Index Number (BIN), a method implemented in BOLD, in which newly submitted and already available sequences clustered in unique BINs using a refined single linkage analysis in which records with high sequence similarity and connectivity are clustered and separated from those with lower similarity and sparse connectivity (Ratnasingham & Hebert, 2013).

Second, we applied four tree‐based approaches, which models speciation along the branches of an inferred phylogenetic: (1) the Single Poisson Tree Processes – sPTP (Zhang et al., 2013), implemented using the PTP online version (http://species.h‐its.org/ptp); (2) the Bayesian Poisson tree process – bPTP (Zhang et al., 2013), also conducted on the PTP web server. Both analyses were conducted with 500,000 MCMC generations and other parameters as default. (3) the Multi‐rate Poisson Tree Processes – mPTP (Kapli et al., 2017), performed with the mPTP web server (https://mcmc‐mptp.h‐its.org/mcmc/), using the multi‐rate Poisson tree process model and following default settings. All PTP analyses (sPTP, mPTP, and bPTP) used the ML trees calculated with Iq‐Tree. (4) the Generalized Mixed Yule Coalescent – GMYC (Pons et al., 2006), performed by submitting the single ultrametric MCC tree resulting from BI obtained from BEAST to the online version of the GMYC software (https://species.h‐its.org/gmyc/), following default single‐threshold (sGMYC). We also tested the multiple‐threshold model (mGMYC); however, it did not perform well, overestimating putative species (data not shown). Similar species estimates with the mGMYC algorithm were also observed in previous studies (Fujisawa & Barraclough, 2013; Schwarzfeld & Sperling, 2015).

### 2.5 Biogeographical analysis

The biogeographical relationships between the studied taxa were implemented using the software PRIMER 7 (Plymouth Routines In Multivariate Ecological Research). In the Neotropical region, we pre‐assigned a set of smaller hierarchical geographical areas (zones), following Morrone et al. (2022), to enable hypothesis testing using non‐metric multidimensional scaling (nMDS) based on the observed species composition determined by our study. The faunal similarity between zones and between larger regions was quantitatively measured using Sørensen similarity index of presence/absence data, and the significance of the geographical groupings was assessed using the ANOSIM test (see Appendices S1 and S2). Species accumulation curves were generated using the vegan package (Oksanen et al., 2017) in the software R (R Core Team, 2021). The analysis involved 100 randomizations. The accumulation curves were constructed using barcode data, considering the presence or absence of each species as delimited by the sGMYC algorithm. This approach was specially chosen to enable a better comparison among samples obtained with different sampling efforts, focusing on the uniqueness of the genetic diversity rather than the abundance of specific species or barcodes.

## 3 RESULTS

### 3.1 Species delimitation

The complete data set consisted of 1492 barcodes, ranging from 312 to 658 bp in length. In total, there were 319 variable sites (48.5%), of which 299 (93.7%) were parsimony informative. Most parsimony informative sites occurred in the third codon position (Table 2). The sequences were heavily AT‐biased, specifically in the third position, which exhibited a combined average AT composition of 89.3% (Table 2). Average intraspecific and interspecific K2P distances for all analyzed *Polypedilum* species were 1.3% and 15.2%, respectively. The barcode gap is an important concept in barcoding studies (Puillandre et al., 2012). It works well when the amount of intraspecific divergence is much smaller than the amount of interspecific variation between species. When this condition is met, a ‘barcoding gap’ exists (Meyer & Paulay, 2005). In general, our data showed clearly larger interspecific than intraspecific divergences, but we still could not observe the expected ‘barcoding gap’ in the pairwise K2P distances. On the contrary, a barcode overlaps between the intraspecific and the interspecific distances was found, which may be attributable to the presence of cryptic species diversity and a few misidentifications. The lack of a gap is usually associated with recently diverged species with little genetic diversification, frequently coupled with incomplete lineage sorting and introgression (Dupuis et al., 2012; Wiemers & Fiedler, 2007).

**TABLE 2:** Variable and informative sites, and average nucleotide composition in the aligned COI gene sequences.

Nucleotide position	Variable sites (%)	Informative sites (%)	T (%)	C (%)	A (%)	G (%)	AT (%)	GC (%)
1st	60.2	21.4	26.9	16.9	28.6	27.6	55.5	44.5
2nd	5.6	3.7	43.1	26.7	13.7	16.5	56.8	43.2
3rd	34.2	74.9	47.2	8.3	42.1	2.4	89.3	10.7
All	48.5%	45.44%	39.0	17.3	28.2	15.5	67.2	32.8

**FIGURE 1:**
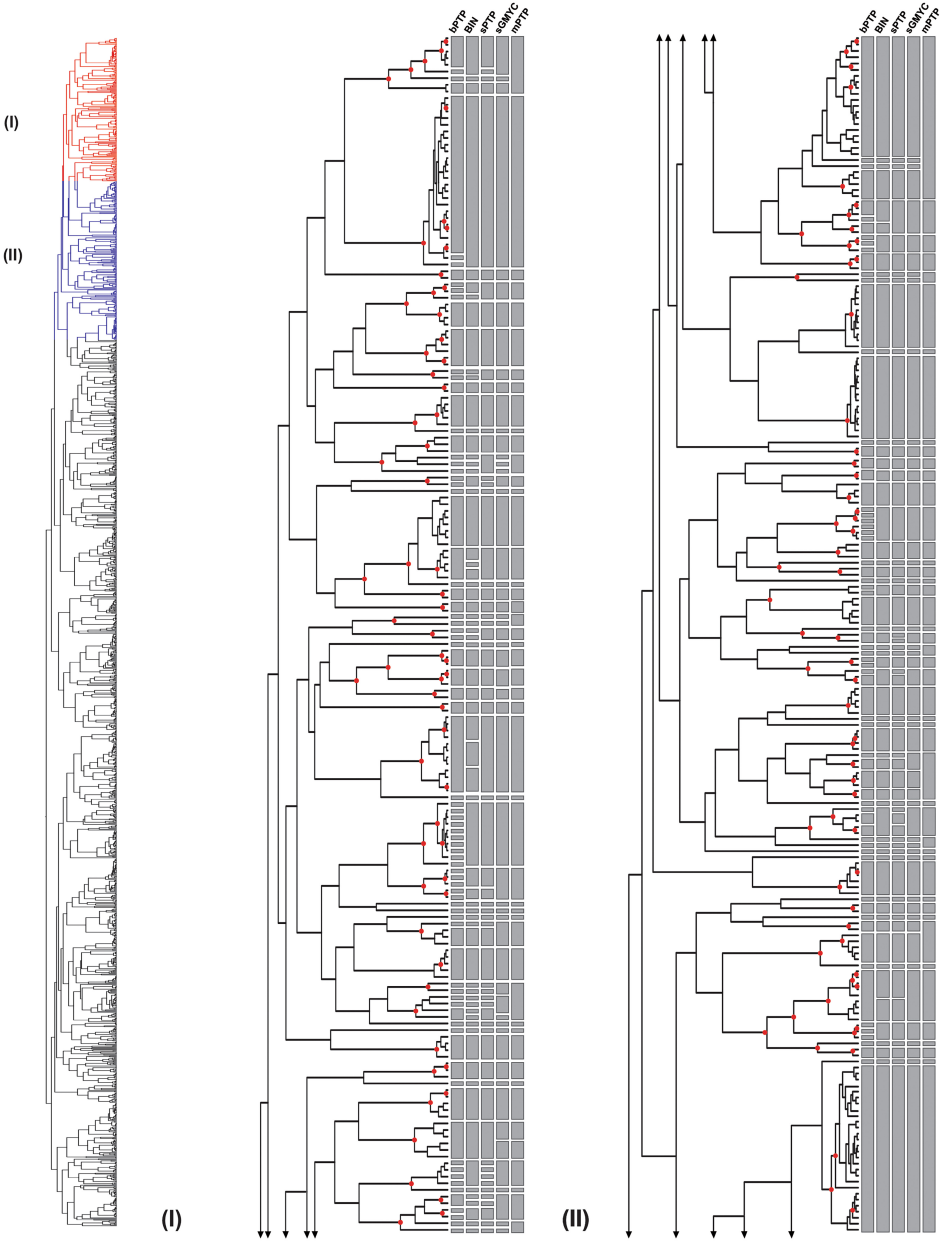
(Part I and II) – Maximum likelihood (ML) phylogeny and species delimitation based on single‐locus DNA barcodes of 1492 specimens of the genus *Polypedilum*, providing an overview on molecular operational taxonomic units (MOTUs) and differences between the distance‐based (BIN) and tree‐based (bPTP, sPTP, sGMYC, and mPTP) methods. Red dots on nodes represent ML bootstrap support >0.95.

**FIGURE 2:**
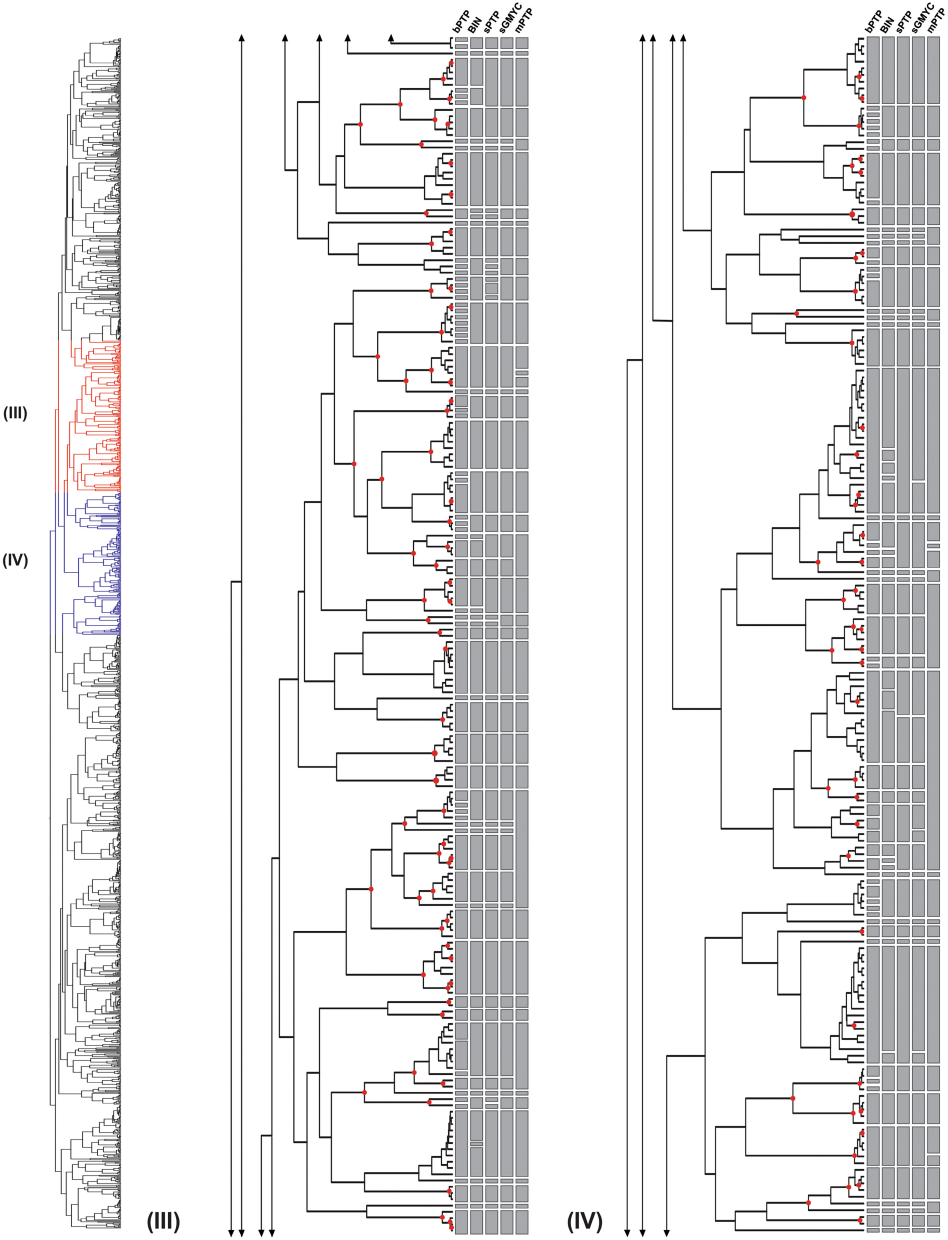
(Part III and IV) – Maximum likelihood (ML) phylogeny and species delimitation based on single‐locus DNA barcodes of 1492 specimens of the genus *Polypedilum*, providing an overview on molecular operational taxonomic units (MOTUs) and differences between the distance‐based (BIN) and tree‐based (bPTP, sPTP, sGMYC, and mPTP) methods (continued from Figure 1). Red dots on nodes represent ML bootstrap support >0.95.

**FIGURE 3:**
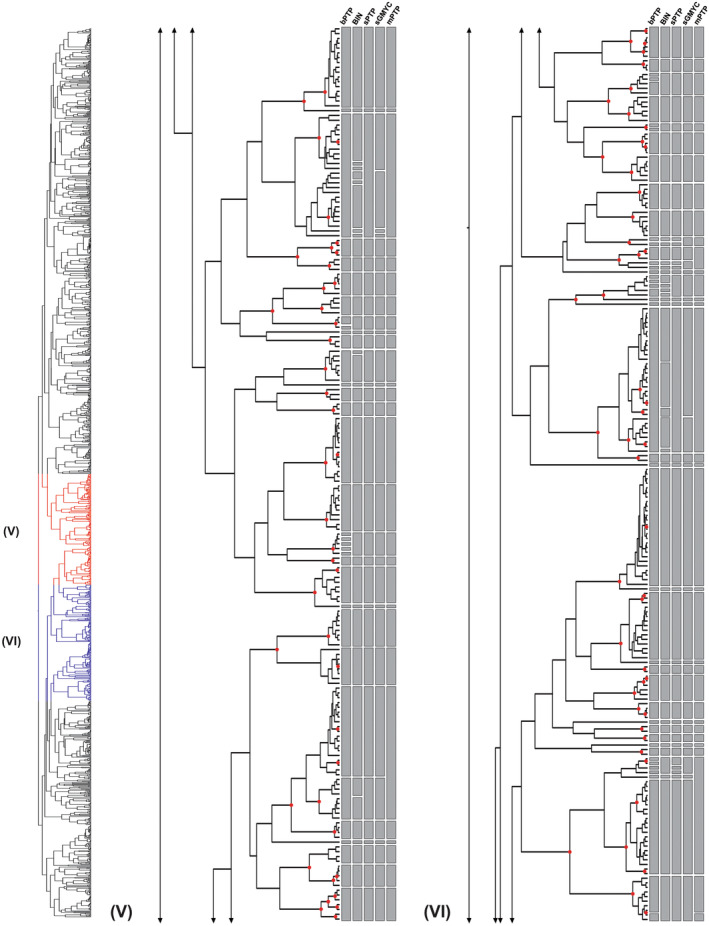
(Part V and VI) – Maximum likelihood (ML) phylogeny and species delimitation based on single‐locus DNA barcodes of 1492 specimens of the genus *Polypedilum*, providing an overview on molecular operational taxonomic units (MOTUs) and differences between the distance‐based (BIN) and tree‐based (bPTP, sPTP, sGMYC, and mPTP) methods (continued from Figure 2). Red dots on nodes represent ML bootstrap support >0.95.

**FIGURE 4:**
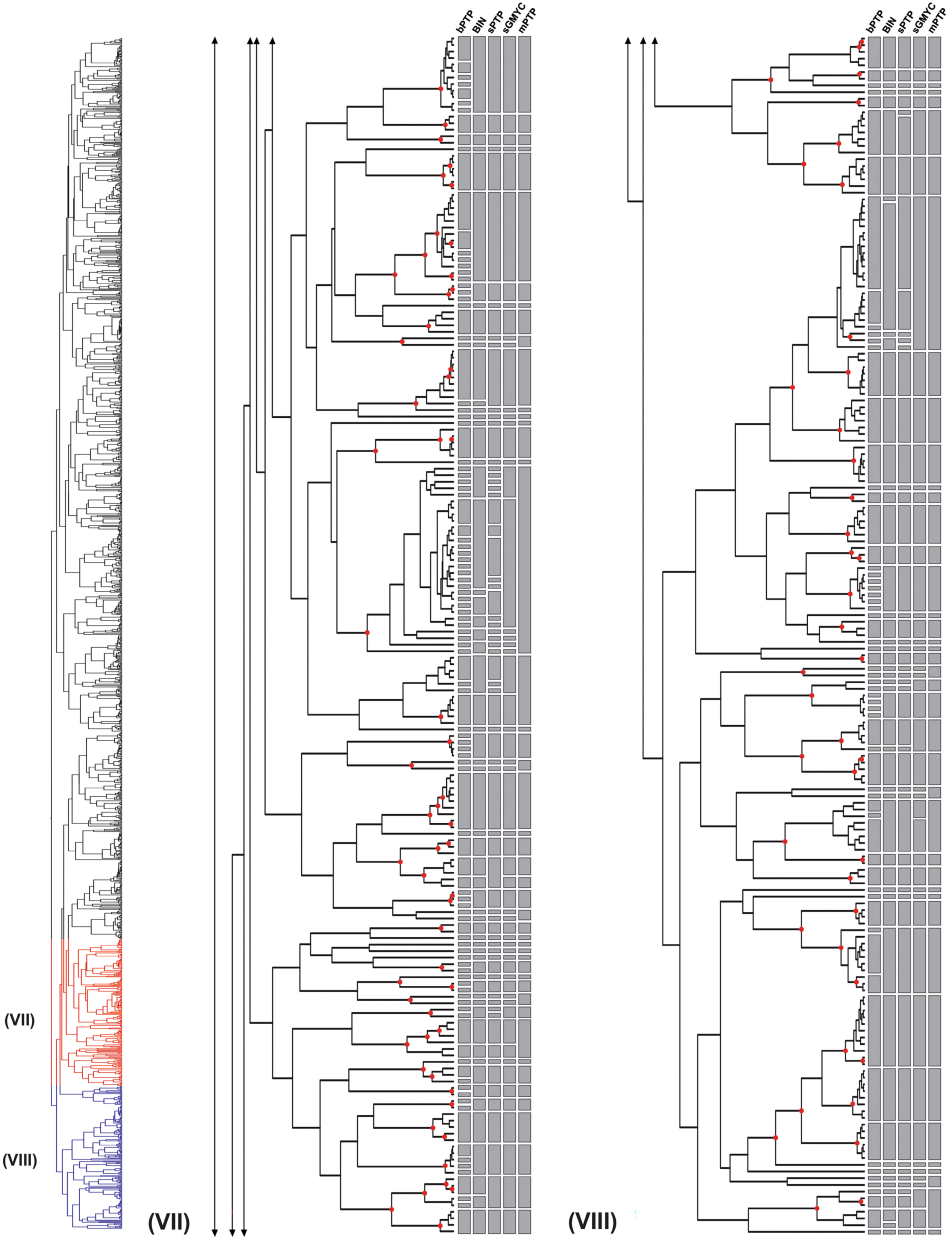
(Part VII and VIII) – Maximum likelihood (ML) phylogeny and species delimitation based on single‐locus DNA barcodes of 1492 specimens of the genus *Polypedilum*, providing an overview on molecular operational taxonomic units (MOTUs) and differences between the distance‐based (BIN) and tree‐based (bPTP, sPTP, sGMYC, and mPTP) methods (continued from Figure 3). Red dots on nodes represent ML bootstrap support >0.95.

Overall, most of the tested methods recovered similar groupings of molecular operational taxonomic units (MOTUs) (Figures 1, 2, 3, 4), with the mPTP method being the most conservative, lumping the sequences into fewer MOTUs, and the bPTP algorithm the most relaxed, splitiing the sequences lumping the sequences into several MOTUs (Table 3). Two out of the three distance‐based methods, ABGD and ASAP, yielded unreliable delimitations with wide confidence intervals, with several clusters not reflecting relationships as understood based on the geographical sampling and others diverging into numerous lineages despite minimal divergence between them. ABGD and ASAP results were therefore not included in Figures 1, 2, 3, 4. The BIN analysis returned a total of 415 MOTUs of which 174 were singleton BINs, 222 concordant BINs, and 19 discordant BINs. In total, 615 sequences of 143 morphospecies were assigned to 179 BINs, including 72 singleton BINs, 83 concordant BINs, and 24 discordant BINs. The unidentified 877 specimens, without binomial names, were assigned to 236 BIN species, including 102 singleton BINs, 118 concordant BINs, and 16 discordant BINs.

**TABLE 3:** Summary of *Polypedilum* species delimitation analyses (bPTP, BIN, sPTP, sGMYC, and mPTP) for each of the major biogeographical regions and zones, as described by Morrone et al. (2022).

Regions	Zones	bPTP	BIN	sPTP	sGMYC	mPTP	Sites
Neotropical	Andean region	25	9	9	8	7	10
	Boreal Brazilian domination	12	11	11	11	10	7
	Chacoan domination	12	11	11	10	9	7
	Pacific domination	2	1	1	1	1	3
	Parana dominion	28	23	24	23	20	7
	South American transition	34	17	26	16	8	2
	Southeastern Amazonia	2	1	1	1	1	1
Afrotropical		11	6	6	6	5	13
Australasian		7	8	6	6	6	12
Nearctic		175	134	138	126	92	291
Oriental		67	65	60	58	51	25
Palearctic		69	69	57	74	51	123
Panamanian		5	5	5	5	5	11
Sino‐Japanese		108	99	95	86	74	86
Total		520	415	411	378	267	598

DNA‐based species delimitation applying bPTP, mPTP, sPTP, and sGMYC resulted in a divergent number of clusters. The single‐threshold general mixed Yule‐coalescent calculations (sGMYC) recovered 378 MOTUs (likelihood ratio: 600.4823, confidence interval: 349–383, threshold time: −0.01053644), while the sPTP model produced a more conservative number of MOTUs (411) compared to the bPTP method, which yielded 520 MOTUs (Table 3). The results from analyses using the multi‐rate PTP (mPTP) model were also comparable to those of the other models, but revealed larger clusters, occasionally joining lineages belonging to different species in a single MOTU (Figures 1, 2, 3, 4). Divergences in the number of clusters generated by the different species delimitation algorithms are caused by erroneously inferred splitting or lumping events (i.e., specimens of one morphospecies were divided or joined into two or more different MOTUs). However, regardless of the method applied, the total number of species delimited in *Polypedilum* in this study is at least twice as high (267–520) as the number of included morphospecies (143, see above).

### 3.2 Biogeography

The following biogeographical analyses were based on the MOTUs (378) delimited by the sGMYC approach (see Discussion section below), while efforts were made to include a broad representation of the species community, variations in sample representation may exist. Overall, the number of species per sampling location varied from a single species to over 15 species across the different sampling sites at the studied biogeographical realms. Just a few of all sampled locations (3.8%) had 5 or more species present, while 203 (53.7%) of the 378 species included were only recorded at a single location. Since collecting methods, sampling sites, protocols, and reporting varied in our dataset, comparisons of global biodiversity between locations are challenging. Numbers of *Polypedilum* species and sampled locations varied between regions (Table 3). Our results indicate that 90.2% of species were recorded only in a single major biogeographical region, while only 36 species spanned two or more of these regions.

**FIGURE 5:**
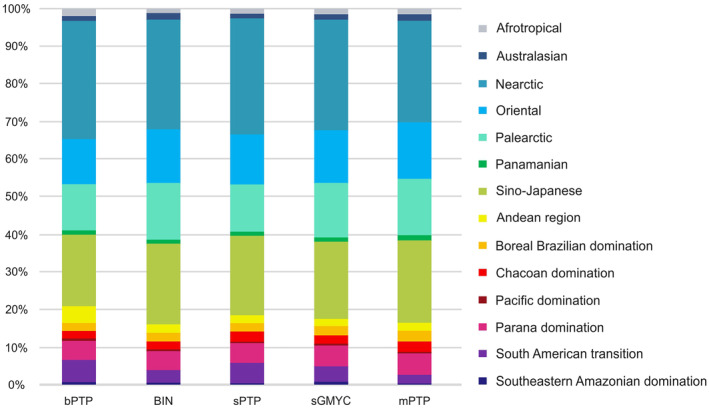
Relative richness (number of species) of putative species of *Polypedilum* based on the different molecular species delimitation methods within the different regions and zones.

The proportion of *Polypedilum* species per region, relative to the total number of species within the genus, exhibited variation across the studied regions (Figure 5). Insufficiently sampled areas (Afrotropical, Australasian, and Panamanian) with low numbers of recorded species present low levels of diversity and are dominated by few species. On the other hand, biogeographical regions exhaustively sampled exhibit high numbers of recorded species with the highest degree of species richness (Nearctic, Palearctic, Oriental, and Sino‐Japanese). The Neotropical region presented moderate levels of *Polypedilum* species diversity, particularly when compared to the neighboring Nearctic region (Figure 5); however, it is noteworthy that although only 6.2% of the sampling sites are located in the Neotropics (mostly in South America), 19.1% of the total number of species occurred in this region. Moreover, based on our results, the Neotropical *Polypedilum* fauna can be considered endemic, since only one unidentified species was also recorded in the Nearctic region.

**FIGURE 6:**
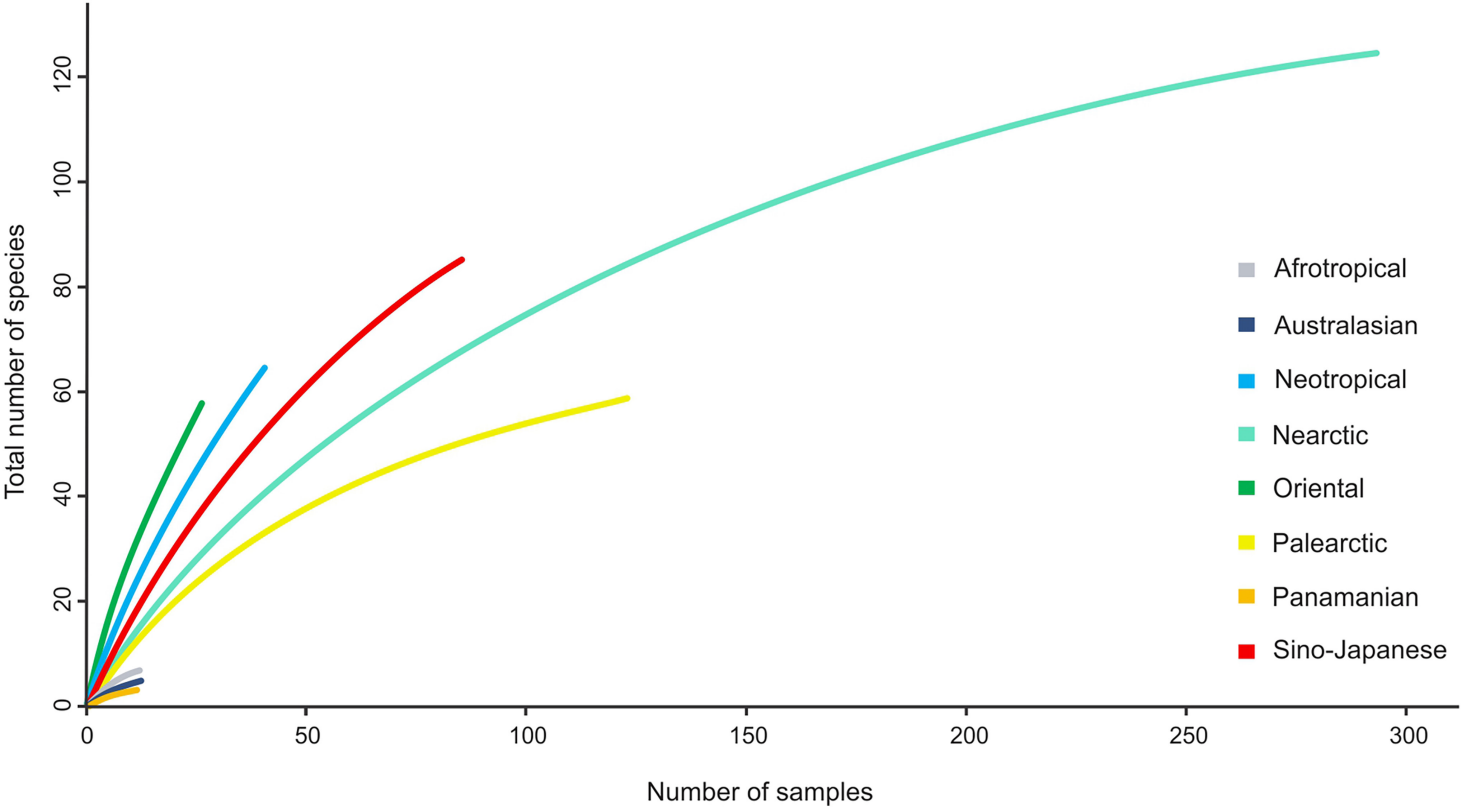
Species accumulation (rarefaction) curves for the putative species of *Polypedilum* estimated by the Generalized Mixed Yule Coalescent (sGMYC) approach within the different biogeographical regions. Samples represent a single collection event.

None of the species accumulation (rarefaction) curves for the biogeographical realms (Figure 6) asymptote, indicating that additional sampling effort could uncover more species in all areas. However, the Nearctic species appear to be approaching a ceiling. We also conduct an additional rarefaction curve analysis by splitting the Palearctic realm into eastern and western regions. However, in this analysis, we did not also observe an asymptotic pattern in the curve (data not shown). The Afrotropical, Australasian, and Panamanian regions exhibited the lowest levels of species richness, while the Nearctic, Palearctic, and Sino‐Japanese regions showed the highest levels of diversity. Interestingly, the species accumulation curves for the Neotropical and Oriental regions, although displaying lower observed species counts, exhibited steeper slopes. This suggests that these regions harbor a greater number of species, which could be revealed by further sampling (Figure 6).

**FIGURE 7:**
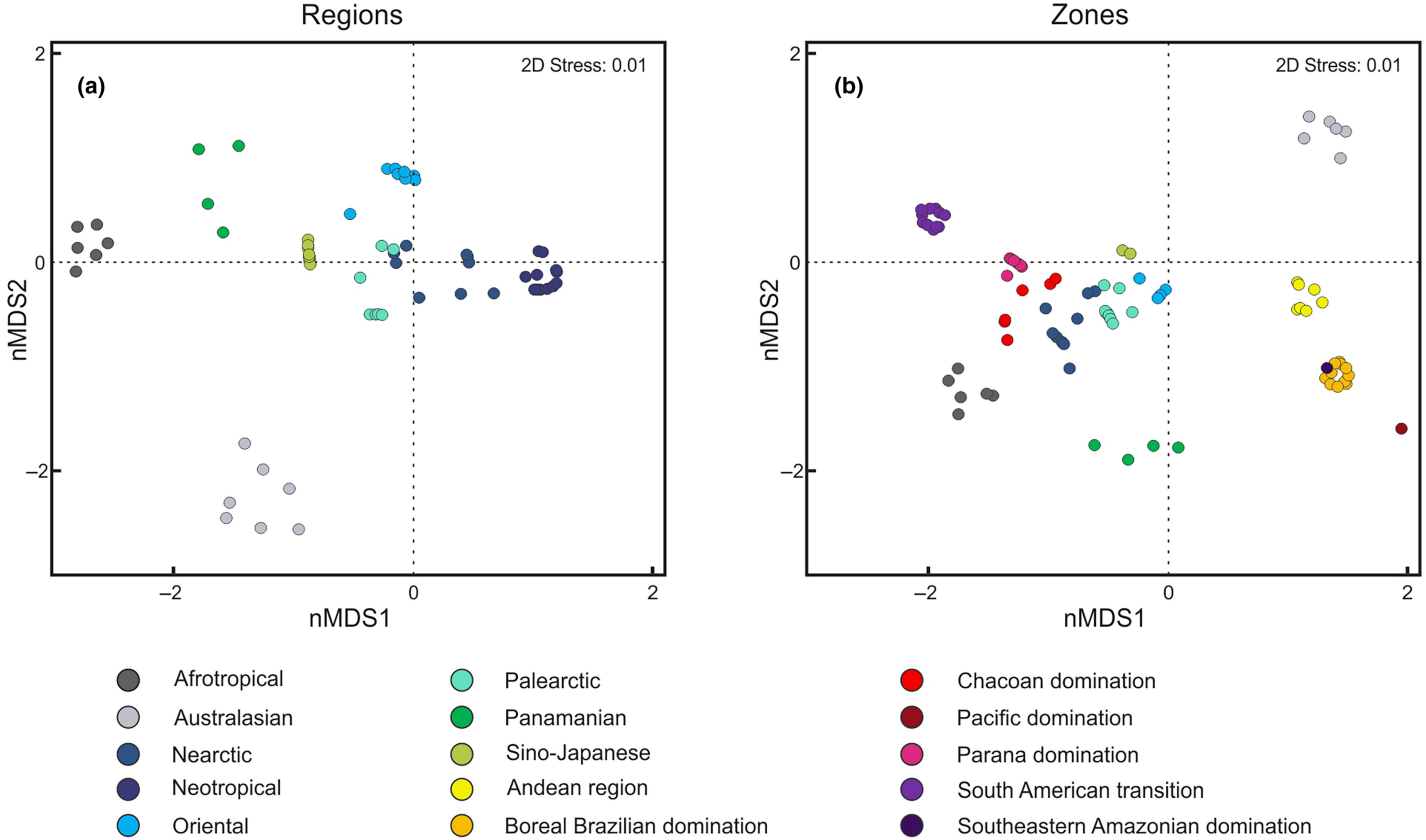
Similarity of putative species of *Polypedilum* estimated by the Generalized Mixed Yule Coalescent (sGMYC) approach within the different biogeographical regions (a) and zones (b), using non‐metric multidimensional scaling (nMDS) using Sørensen similarity index of presence/absence applied to species data.

Biogeographical realm patterns across the entire assembly (Figure 7a) showed distinct groupings for Afrotropical and Australasian, while the ANOSIM (see Appendix S1) and nMDS results show some overlap between Nearctic and Palearctic regions. The Neotropical *Polypedilum* fauna despite the closeness to the Nearctic region presents distinct clustering. The different Neotropical zones compose distinct well‐supported groups (Figure 7b), with some degree of overlap between Southeastern Amazonia and Boreal Brazilian domination zones. In particular, the Palearctic region appears to display affinities for both the Nearctic and Oriental region (Figure 7b).

## 4 DISCUSSION

### 4.1 Species delimitation

One of the objectives of this study was to explore the utility of a large‐scale single‐locus DNA barcode analysis of the genus *Polypedilum* to investigate its molecular diversity and compare the adequacy of molecular species delimitation approaches. Our results suggest that tree‐based algorithms are more suitable than distanced‐based because they are able to integrate evolutionary theory, not requiring arbitrary thresholds (Schwarzfeld & Sperling, 2015). In our study, ABGD and ASAP produced unreasonable delimitations, not consistently proposing species hypotheses. These approaches are known to over‐lump, performing poorly on more speciose datasets such as ours, whereas the success rate increases remarkably for small populations (Dellicour & Flot, 2015, 2018). It is worth noting that the performance of these methods can vary depending on the dataset characteristics and analytical strategies employed. For instance, Song et al. (2018) reported successful outcomes with ABGD when analyzing a *Polypedilum* COI barcode dataset. In contrast to ABGD and ASAP's over‐lumping, the Barcode Index Number (BINs) method, assigned by BOLD, is known to oversplit species numbers due to the low intracluster distance (2.2%) at the initial clustering step of RESL algorithm (Ratnasingham & Hebert, 2013). Similar results were found by Song et al. (2018), when applying the BIN system also to delimit *Polypedilum* species, mostly from East Asia.

Among the drawbacks of distance‐based methods, such as ABGD and ASAP, is the lack of a fixed threshold, which may be universally applicable to all taxa (Yang & Rannala, 2017). Different taxonomic groups can exhibit varying levels of genetic divergence within and between species, posing a challenge in establishing a single threshold that accurately distinguishes species. This limitation highlights the need for alternative approaches in species delimitation. By employing multiple analytical approaches, the accuracy and reliability of species assignments can be assessed from different perspectives and account for the inherent variability in genetic distances among taxa. This approach allows for a more comprehensive evaluation of species delimitation and increases the likelihood of capturing the true diversity within a genus or taxonomic group. Another downside of distance‐based approaches is that they do not consider evolutionary relationships into their algorithms (Kapli et al., 2017). On the other hand, tree‐based methods, including bPTP, sGMYC, and PTP, are not influenced by such thresholds, often performing better as evolutionary relationships and branch length distributions are incorporated (Song et al., 2018).

When applied to our dataset, we observed that tree‐based methods, particularly sGMYC and PTP, exhibited greater congruence with the morphological species concept compared to distance‐based methods. This suggests that the results obtained from sGMYC and PTP were more consistent with the traditionally recognized species based on morphological characteristics. For example, the Poisson Tree Process (PTP) method relies on the distribution of branch lengths in the gene tree in order to identify species status (Zhang et al., 2013). The tree and branch lengths are inferred from a sequence alignment using maximum likelihood and then treated as lacking errors (Rannala & Yang, 2020). In our study, there was a large difference between recovered MOTUs among the PTP methods. There was a 109 MOTU difference between results based on the bPTP and sPTP methods. mPTP was the most conservative and commonly underestimated species by lumping singleton species, represented in our tree by isolated branches, into MOTUs. Along with our results, other studies have found that the mPTP algorithm leads to a lower number of recovered species when compared with other approaches (e.g., da Silva et al., 2018; Parslow et al., 2021).

The sGMYC analysis based on a single gene revealed the presence of 378 MOTUs. This species‐delimitation algorithm relies on the priors and parameters used to construct the ultrametric tree (Ceccarelli et al., 2012), and tends to overestimate species diversity compared to other methods (Kekkonen & Hebert, 2014; Miralles & Vences, 2013; Paz & Crawford, 2012; Talavera et al., 2013). In our study, the sGMYC method seems to be the most accurate since it recovered substantially fewer putative species than the bPTP and sPTP analyses despite its hypothesized oversplitting. Moreover, the sGMYC approach has been suggested to suit datasets with large numbers of singleton taxa (Talavera et al., 2013), which is what we observe for *Polypedilum*. Based on the aforementioned considerations, we chose the putative species delimited by the sGMYC method as the basis for the biogeographical analyses.

### 4.2 Biogeography

The level of taxonomic diversity present in an environment can be quantified by either enumerating numbers of species or estimating evolutionary divergences among species through genetic analyses (Webb, 2000). Moreover, besides the number of individuals sampled, the size of the local species pool, the evenness of species abundances in the community, size and environmental heterogeneity of the area, and the status of taxonomic understanding of the taxa investigated are parameters essential to the accuracy of estimates of taxonomic diversity (Antonelli et al., 2018). Although most measures of alpha and beta diversity rely on species numbers, DNA sequence data may provide an evolutionary framework to diversity estimates (Hebert et al., 2016). In this sense, genetic measures may also be used to evaluate species boundaries when compared with species richness in the same communities. Additionally, DNA barcodes can be used for species delimitation, assisting in documenting new species and identifying targeted habitats for conservation (Faith, 1992, 2008). In geographic regions especially known for their unique lineages of organisms, biological diversity determined with DNA barcode sequence data can be essential for comparing diversity and establishing protected areas across the landscape (Hobern, 2021; Shapcott et al., 2015).

Numerical species delimitation methods require species to be sufficiently sampled (Dopheide et al., 2019) across geographical ranges to improve their ability to correctly delimit species (Parslow et al., 2021). In practice, this is a challenging task when it comes to *Polypedilum* due to its known worldwide diversity of ca. 440 described species and the expected number of undescribed species. Although recent taxonomic studies of regional fauna have been conducted (Song et al., 2016, 2018), particularly in East Asia, there are several regions that need modern taxonomic treatments, for example, Australia, Africa, and South America. Therefore, it is difficult to determine the degree of sampling completeness of *Polypedilum* caused by the potentially large number of undescribed species. In the current study, many of the biogeographical differences in recorded species numbers can be ascribed to different sampling efforts and methods between regions. Usually, knowledge of species distributions and diversity patterns is strongly concentrated toward areas which are more easily accessible by roads, rivers, and research stations (Antonelli et al., 2018). This fact is evident in our investigation, as though we included all publicly available COI sequences for *Polypedilum* in BOLD, there was a bias toward Nearctic (33.2%) and Sino‐Japanese (23.2%) taxa, with a reduced representation of Afrotropical (1.6%), Australasian (1.6%) and Panamanian (1.3%) species, regions known for receiving less investment for research in Chironomidae.

Much of what we need to comprehend about biodiversity can be undertaken as a matrix of the presence or abundance of multiple species across time and space (Hobern, 2021). That said, plotting species accumulation curves permit researchers to measure and compare diversity across populations or to assess the benefits of further sampling (Deng et al., 2015). In our study, the rarefaction curve analysis suggests that even when randomization sampling methods are considered there are regional differences in species richness. Noticeably, the most species‐rich regions were the Nearctic and Sino‐Japanese regions. This came as a little surprise, since we expected the Palearctic region also to be among the most specious biogeographical areas, due to the high number of *Polypedilum* sequences available in BOLD and the numerous studies performed on the family Chironomidae in this area. Although we used species accumulation curves to indicate the pattern of sequence accumulation within the current study, they are not expected to represent the accurate diversity of each region, as they are not based on actual random sampling (Schwarzfeld & Sperling, 2015).

In the Neotropical region, the biogeographical patterns of chironomids are shaped by a multitude of factors, including but not limited to habitat heterogeneity, climate conditions, geological history, and dispersal abilities of the species (Antonelli, 2022). While our findings highlight the sharing of species between distinct communities in the Palearctic and Nearctic regions, indicating a complex biogeographical scenario, which is also reported by Ekrem et al. (2018) and Marusik and Koponen (2005), the Neotropical region presents its own unique characteristics. The high biodiversity and diverse ecological conditions of the Neotropics contribute to distinct patterns of chironomid distribution. Furthermore, when considering the overlapping Nearctic and Palearctic *Polypedilum* fauna, it is likely that numerous faunal interchanges that took place across the Bering land bridge in the past (135,000–70,000 YBP), primarily involving large, cold‐tolerant species (Rodríguez et al., 2006). Aerial plankton observations of chironomids (Cotoras & Zumbad, 2020; Gressitt et al., 1960; Hardy & Milne, 1938) and potential long‐distance dispersal support trans‐Atlantic distribution patterns in *Polypedilum* (Ekrem et al., 2018). Finally, despite the altitudinal barrier of the Himalayas, species overlap was also recorded between Palearctic and Oriental fauna, as seen in butterflies (Larsen, 1984). The majority of chironomid species (54.9%) are recorded at a single location, suggesting small number of widely distributed species driving the regional and larger‐scale biogeographical patterns. The high number of species recorded only once reflects the understudied nature of these taxa (Velasco‐Castrillón et al., 2014; Zhang et al., 2018).

In the present study, the Neotropical region as one of the lesser studied regions with 73 species recorded from 48 localities (Neotropical + Panamanian) exhibited a higher species richness than that of the Palearctic and Oriental realms. Moreover, despite the Neotropical fauna has some boreal components, such as *Polypedilum beckae* (Sublette, 1964) and *Paralauterborniella nigrohalteralis* (Malloch, 1915), from the adjacent Nearctic fauna according to Silva et al. (2015), our study's results support our hypothesis of notable differences in species composition between the *Polypedilum* fauna in South America and the neighboring regions. Only a single unidentified species spanned from the Neotropical region to the Nearctic region, recorded in Argentina and Mexico, which confirms our expectations of high levels of endemism and richness of *Polypedilum* species in the Neotropical region. The outstanding biodiversity there, when compared to other major biotic realms (Antonelli & Sanmartín, 2011; Lundberg et al., 2000) can be attributed to a complex process in which palaeo‐geographical and palaeoclimatic forces have been constantly interacting and new species have originated continuously in that area since the late Eocene/early Oligocene (Grund, 2006; Rull, 2008). As such, South America is paramount for research on the origin of biological diversity. Finally, some neotropical areas are under manifest danger of biodiversity loss (Antonelli, 2022). Our study shows that DNA‐based species delimitation approaches can be used in rapid biodiversity estimates of poorly known taxonomic groups so these can be utilized as basis for biodiversity conservation strategies, and to unravel biogeographical patterns at both local and global scales.

### 4.3 Conclusion: Implications of DNA barcoding to accelerate biogeography research

As different analytical methods have different theoretical foundations, it is advisable to test a wide variety of approaches of species delimitation and to favor patterns that are congruent across the results. Moreover, the contrast of different methods helps to comprehend their propensity to either split or lump clusters. We evaluated some approaches for species delimitation in the genus *Polypedilum* through single‐locus DNA barcodes and found the sGMYC as the method more adequate to estimate putative species on our dataset. Our results highlight *Polypedilum* as species‐rich genus, yet incompletely documented, which implies in the need of increased taxon sampling, across geographical ranges, and the use of additional molecular data for greater resolution when using molecular species delimitation approaches for the group. Quantitative species delimitation methods are sensitive to sampling effort. Since communities typically contain several species that are locally rare, observed species richness provides just an underestimate of the diversity actually present, except if the community is thoroughly sampled. Therefore, a reference COI sequence library derived from expert‐identified reference material is fundamental to assign organisms into species by matching the sequence of an unknown sample to the reference library. Our hypothesis that there would be substantial differences in species composition between the *Polypedilum* fauna in South America and other neighboring regions, particularly the Nearctic region, was confirmed. The Neotropical region exhibited high levels of endemism and richness for *Polypedilum* species. Despite major advances in our understanding of Neotropical biodiversity in recent years, several questions remain to be answered: When did the region reach globally outstanding levels of species richness? Why do nearly all groups of organisms have more species in the Neotropical region? What factors contribute to the higher species count in the Neotropical region across various organism groups? What are the underlying drivers of latitudinal patterns in biodiversity? When did the currently observed species split from their most recent common ancestors? Further biological and geological data, associated with the integration of different DNA‐based methods for estimating species richness, will advance the field of natural history and increase our ability to make knowledge‐based decisions in conservation issues. The integration of biodiversity genomics in biogeography science therefore represents a major scientific priority.

## AUTHOR CONTRIBUTIONS

**Fabio Laurindo da Silva:** Conceptualization (equal); data curation (equal); formal analysis (lead); funding acquisition (lead); investigation (lead); methodology (lead); project administration (lead); writing – original draft (equal); writing – review and editing (equal). **Luiz Carlos Pinho:** Data curation (equal); writing – review and editing (equal). **Elisabeth Stur:** Conceptualization (equal); writing – original draft (equal); writing – review and editing (equal). **Silvio Shigueo Nihei:** Project administration (supporting); writing – review and editing (supporting). **Torbjørn Ekrem:** Conceptualization (equal); writing – original draft (lead); writing – review and editing (equal).

## CONFLICT OF INTEREST STATEMENT

The authors declare no conflict of interest.

### OPEN RESEARCH BADGES

This article has earned Open Data, Open Materials and Preregistered Research Design badges. Data, materials and the preregistered design and analysis plan are available at http://www.boldsystems.org/index.php/Public_SearchTerms?query=<RRPOL>.

## Supporting information


Appendix S1.

## Data Availability

The detailed specimen records and sequence information, including trace files, are freely available in BOLD through the dataset ‘DS‐RRPOL, Reduced Records of *Polypedilum* (Diptera: Chironomidae)’ with DOI: https://doi.org/10.5883/DS‐RRPOL. Significant ANOSIM results per realm and region are given in Appendices S1 and S2.
