# Supplementary material for: DNA barcodes provide insights into the diversity and biogeography of the non‐biting midge Polypedilum (Diptera, Chironomidae) in South America
Source: Ecol Evol. 2023 Oct 11;13(10):e10602. doi: 10.1002/ece3.10602 (PMC10568203; doi:10.1002/ece3.10602)
Supplement: Supplementary file 1 — Appendix S1. [file ECE3-13-e10602-s001.docx]

**Appendix S1 – Significant ANOSIM results per realm.**

Global Test

Sample statistic (R): 0.993

Significance level of sample statistic: 0.1%

Number of permutations: 999 (Random sample from a large number)

Number of permuted statistics greater than or equal to R: 0

| **Groups** | **R**  **Statistic** | **Significance**  **Level %** | **Possible**  **Permutations** | **Actual**  **Permutations** | **Number >=**  **Observed** |
| --- | --- | --- | --- | --- | --- |
| Afrotropical, Australasian | 1 | 0.2 | 462 | 462 | 1 |
| Afrotropical, Nearctic | 1 | 0.1 | Very large | 999 | 0 |
| Afrotropical, Neotropical | 1 | 0.1 | 170230452 | 999 | 0 |
| Afrotropical, Oriental | 1 | 0.1 | 50063860 | 999 | 0 |
| Afrotropical, Palearctic | 1 | 0.1 | 28989675 | 999 | 0 |
| Afrotropical, Panamanian | 1 | 0.5 | 210 | 210 | 1 |
| Afrotropical, Sino-Japanese | 1 | 0.1 | 109453344 | 999 | 0 |
| Australasian, Nearctic | 1 | 0.1 | Very large | 999 | 0 |
| Australasian, Neotropical | 1 | 0.1 | 170230452 | 999 | 0 |
| Australasian, Oriental | 1 | 0.1 | 50063860 | 999 | 0 |
| Australasian, Palearctic | 1 | 0.1 | 28989675 | 999 | 0 |
| Australasian, Panamanian | 1 | 0.5 | 210 | 210 | 1 |
| Australasian, Sino-Japanese | 1 | 0.1 | 109453344 | 999 | 0 |
| Nearctic, Neotropical | 0.998 | 0.1 | Very large | 999 | 0 |
| Nearctic, Oriental | 0.999 | 0.1 | Very large | 999 | 0 |
| Nearctic, Palearctic | 0.983 | 0.1 | Very large | 999 | 0 |
| Narcotic, Panamanian | 0.998 | 0.1 | 10009125 | 999 | 0 |
| Nearctic, Sino-Japanese | 0.999 | 0.1 | Very large | 999 | 0 |
| Neotropical, Oriental | 1 | 0.1 | Very large | 999 | 0 |
| Neotropical, Palearctic | 1 | 0.1 | Very large | 999 | 0 |
| Neotropical, Panamanian | 1 | 0.1 | 971635 | 999 | 0 |
| Neotropical, Sino-Japanese | 1 | 0.1 | Very large | 999 | 0 |
| Oriental, Palearctic | 0.98 | 0.1 | Very large | 999 | 0 |
| Oriental, Panamanian | 1 | 0.1 | 424270 | 999 | 0 |
| Oriental, Sino-Japanese | 0.956 | 0.1 | Very large | 999 | 0 |
| Palearctic, Panamanian | 1 | 0.1 | 292825 | 999 | 0 |

**Appendix S2 – Significant ANOSIM results per region.**

Global Test

Sample statistic (Global R): 0.452

Significance level of sample statistic: 0.1%

Number of permutations: 999 (Random sample from a large number)

Number of permuted statistics greater than or equal to Global R: 0

| **Groups** | **R**  **Statistic** | **Significance**  **Level %** | **Possible**  **Permutations** | **Actual**  **Permutations** | **Number >=**  **Observed** |
| --- | --- | --- | --- | --- | --- |
| Afrotropical, Australasian | 1 | 0.2 | 462 | 462 | 1 |
| Afrotropical, Nearctic | 1 | 0.1 | Very large | 999 | 0 |
| Afrotropical, Oriental | 1 | 0.1 | 50063860 | 999 | 0 |
| Afrotropical, Palearctic | 1 | 0.1 | 28989675 | 999 | 0 |
| Afrotropical, Panamanian | 1 | 0.5 | 210 | 210 | 1 |
| Afrotropical, Sino-Japanese | 1 | 0.1 | 109453344 | 999 | 0 |
| Afrotropical, Andean region | 1 | 0.1 | 1716 | 999 | 0 |
| Afrotropical, Boreal Brazilian domination | 1 | 0.1 | 12376 | 999 | 0 |
| Afrotropical, Chacoan domination | 1 | 0.1 | 5005 | 999 | 0 |
| Afrotropical, Pacific domination | 1 | 14.3 | 7 | 7 | 1 |
| Afrotropical, Parana dominion | 1 | 0.1 | 296010 | 999 | 0 |
| Afrotropical, South American transition | 1 | 0.1 | 134596 | 999 | 0 |
| Australasian, Narcotic | 1 | 0.1 | Very large | 999 | 0 |
| Australasian, Oriental | 1 | 0.1 | 50063860 | 999 | 0 |
| Australasian, Palearctic | 1 | 0.1 | 28989675 | 999 | 0 |
| Australasian, Panamanian | 1 | 0.5 | 210 | 210 | 1 |
| Australasian, Sino-Japanese | 1 | 0.1 | 109453344 | 999 | 0 |
| Australasian, Andean region | 1 | 0.2 | 1716 | 999 | 1 |
| Australasian, Boreal Brazilian domination | 1 | 0.1 | 12376 | 999 | 0 |
| Australasian, Chacoan domination | 1 | 0.1 | 5005 | 999 | 0 |
| Australasian, Pacific domination | 1 | 14.3 | 7 | 7 | 1 |
| Australasian, Parana dominion | 1 | 0.1 | 296010 | 999 | 0 |
| Australasian, South American transition | 1 | 0.1 | 134596 | 999 | 0 |
| Nearctic, Oriental | 0.999 | 0.1 | Very large | 999 | 0 |
| Nearctic, Palearctic | 0.983 | 0.1 | Very large | 999 | 0 |
| Nearctic, Panamanian | 0.998 | 0.1 | 10009125 | 999 | 0 |
| Nearctic, Sino-Japanese | 0.999 | 0.1 | Very large | 999 | 0 |
| Nearctic, Andean region | 1 | 0.1 | Very large | 999 | 0 |
| Nearctic, Boreal Brazilian domination | 1 | 0.1 | Very large | 999 | 0 |
| Nearctic, Chacoan domination | 0.998 | 0.1 | Very large | 999 | 0 |

**Appendix S2 – Continued.**

| **Groups** | **R**  **Statistic** | **Significance**  **Level %** | **Possible**  **Permutations** | **Actual**  **Permutations** | **Number >=**  **Observed** |
| --- | --- | --- | --- | --- | --- |
| Nearctic, Pacific domination | 1 | 0.8 | 123 | 123 | 1 |
| Nearctic, Parana dominion | 1 | 0.1 | Very large | 999 | 0 |
| Nearctic, South American transition | 1 | 0.1 | Very large | 999 | 0 |
| Oriental, Palearctic | 0.98 | 0.1 | Very large | 999 | 0 |
| Oriental, Panamanian | 1 | 0.1 | 424270 | 999 | 0 |
| Oriental, Sino-Japanese | 0.956 | 0.1 | Very large | 999 | 0 |
| Oriental, Andean region | 1 | 0.1 | 436270780 | 999 | 0 |
| Oriental, Boreal Brazilian domination | 1 | 0.1 | Very large | 999 | 0 |
| Oriental, Chacoan domination | 1 | 0.1 | Very large | 999 | 0 |
| Oriental, Pacific domination | 1 | 1.8 | 55 | 55 | 1 |
| Oriental, Parana dominion | 1 | 0.1 | Very large | 999 | 0 |
| Oriental, South American transition | 1 | 0.1 | Very large | 999 | 0 |
| Palearctic, Panamanian | 1 | 0.1 | 292825 | 999 | 0 |
| Palearctic, Sino-Japanese | 0.985 | 0.1 | Very large | 999 | 0 |
| Palearctic, Andean region | 1 | 0.1 | 231917400 | 999 | 0 |
| Palearctic, Boreal Brazilian domination | 1 | 0.1 | Very large | 999 | 0 |
| Palearctic, Chacoan domination | 1 | 0.1 | Very large | 999 | 0 |
| Palearctic, Pacific domination | 1 | 2 | 50 | 50 | 1 |
| Palearctic, Parana dominion | 1 | 0.1 | Very large | 999 | 0 |
| Palearctic, South American transition | 1 | 0.1 | Very large | 999 | 0 |
| Panamanian, Sino-Japanese | 1 | 0.1 | 720720 | 999 | 0 |
| Panamanian, Andean region | 1 | 0.3 | 330 | 330 | 1 |
| Panamanian, Boreal Brazilian domination | 1 | 0.2 | 1365 | 999 | 1 |
| Panamanian, Chacoan domination | 1 | 0.1 | 715 | 715 | 1 |
| Panamanian, Pacific domination | 1 | 20 | 5 | 5 | 1 |
| Panamanian, Parana dominion | 1 | 0.1 | 12650 | 999 | 0 |
| Panamanian, South American transition | 1 | 0.1 | 7315 | 999 | 0 |
| Sino-Japanese, Andean region | 1 | 0.1 | Very large | 999 | 0 |
| Sino-Japanese, Boreal Brazilian domination | 1 | 0.1 | Very large | 999 | 0 |
| Sino-Japanese, Chacoan domination | 1 | 0.1 | Very large | 999 | 0 |
| Sino-Japanese, Pacific domination | 1 | 1.6 | 63 | 63 | 1 |
| Sino-Japanese, Parana dominion | 1 | 0.1 | Very large | 999 | 0 |
| Sino-Japanese, South American transition | 1 | 0.1 | Very large | 999 | 0 |
| Andean region, Boreal Brazilian domination | 1 | 0.1 | 31824 | 999 | 0 |
| Andean region, Chacoan domination | 1 | 0.2 | 11440 | 999 | 1 |

**Appendix S2 – Continued.**

| **Groups** | **R**  **Statistic** | **Significance**  **Level %** | **Possible**  **Permutations** | **Actual**  **Permutations** | **Number >=**  **Observed** |
| --- | --- | --- | --- | --- | --- |
| Andean region, Pacific domination | 1 | 12.5 | 8 | 8 | 1 |
| Andean region, Parana dominion | 1 | 0.1 | 1184040 | 999 | 0 |
| Andean region, South American transition | 1 | 0.1 | 480700 | 999 | 0 |
| Boreal Brazilian domination, Chacoan domination | 1 | 0.1 | 167960 | 999 | 0 |
| Boreal Brazilian domination, Pacific domination | 1 | 8.3 | 12 | 12 | 1 |
| Boreal Brazilian domination, Parana dominion | 1 | 0.1 | 129024480 | 999 | 0 |
| Boreal Brazilian domination, South American transition | 1 | 0.1 | 34597290 | 999 | 0 |
| Chacoan domination, Pacific domination | 1 | 10 | 10 | 10 | 1 |
| Chacoan domination, Parana dominion | 0.987 | 0.1 | 14307150 | 999 | 0 |
| Chacoan domination, South American transition | 1 | 0.1 | 4686825 | 999 | 0 |
| Pacific domination, Parana dominion | 1 | 4.5 | 22 | 22 | 1 |
| Pacific domination, South American transition | 1 | 5.3 | 19 | 19 | 1 |
| Parana dominion, South American transition | 1 | 0.1 | Very large | 999 | 0 |
